# Case Report: Emphysematous Pyelonephritis With a Congenital Giant Ureterocele

**DOI:** 10.3389/fped.2021.775468

**Published:** 2021-11-26

**Authors:** Hiroyuki Kitano, Keisuke Hieda, Hiroki Kitagawa, Yusuke Nakaoka, Yumiko Koba, Kohei Ota, Norifumi Shigemoto, Tetsutaro Hayashi, Seiya Kashiyama, Jun Teishima, Nobuaki Shime, Hiroki Ohge, Nobuyuki Hinata

**Affiliations:** ^1^Department of Urology, Graduate School of Biomedical and Health Sciences, Hiroshima University, Hiroshima, Japan; ^2^Department of Infectious Diseases, Hiroshima University Hospital, Hiroshima, Japan; ^3^Department of Clinical Practice and Support, Hiroshima University Hospital, Hiroshima, Japan; ^4^Department of Emergency and Critical Care Medicine, Graduate School of Biomedical and Health Sciences, Hiroshima University, Hiroshima, Japan

**Keywords:** emphysematous pyelonephritis, congenital ureterocele, children, *Actinotignum schaalii*, *Peptoniphilus asaccharolyticus*

## Abstract

A 14-year-old girl noticed malodorous urine and experienced left flank pain. The patient was presented to our hospital with gradually increasing pain. She had no underlying disease but had a history of pain on micturition for several days. Hematologic examination indicated low white blood cell and platelet counts and a high serum lactate level. Computed tomography showed that a part of the parenchyma of the left kidney had poor contrast and was deteriorated, with fluid and gas retention from the perirenal region to the retroperitoneal cavity. A left hydroureter and large ureterocele were observed in the bladder. She was diagnosed with emphysematous pyelonephritis (EPN) with a giant congenital ureterocele. Vasopressors and blood transfusion failed to maintain normal circulatory dynamics, and an open left nephrectomy and transurethral ureterocele fenestration were performed. The excised outer portion of the left kidney was dissolved by the infection and replaced with blood clots and necrotic tissue. Matrix-assisted laser desorption/ionization time-of-flight mass spectrometry identified the inflammatory, gas-producing bacteria *Actinotignum schaalii, Peptoniphilus asaccharolyticus*, and *Actinomyces odontolyticus*. Meropenem was administered for 4 days postoperatively and then de-escalated to sulbactam/ampicillin for another 10 days. The patient was discharged on day 17 of hospitalization, and the postoperative course remained favorable. EPN is extremely rare in pediatric patients, and it is believed that nephrectomy is sometimes necessary if the patient does not have normal circulatory dynamics despite the use of catecholamines.

## Introduction

Emphysematous pyelonephritis (EPN) is an acute, severe necrotizing infection of the renal parenchyma, its surrounding tissue, and the urinary tract, and results in gas accumulation in the kidney or perinephric tissue (1, 2). EPN is a rare disease with a mortality rate of ~20% (3, 4). In adults, diabetes mellitus, urinary tract obstruction, and immune-incompetence predispose patients to EPN (2, 5), and *Escherichia coli* is the most common pathogenic organism, accounting for 43–70% of cases (2, 3). However, the pathogenesis of EPN in children remains unclear, partially due to a paucity of reports on EPN in children.

The clinical course of EPN can be severe and life-threatening without prompt diagnosis and treatment. Computed tomography helps prompt diagnosis by revealing renal gas accumulation, and surgical intervention and antibiotic therapy are the primary therapeutic options (3, 4). To date, very few cases of EPN have been reported in pediatric patients (6–12). Herein, we report a case of EPN in a child with a congenital giant ureterocele. Inflammatory bacteria, including *Actinotignum schaalii, Peptoniphilus asaccharolyticus*, and *Actinomyces odontolyticus*, were identified; to the best our knowledge, this is the first report of EPN caused by these bacteria in a child. A review of the relevant literature regarding pediatric EPN was also conducted.

## Case Description

A 14-year-old girl was presented to our hospital with a complaint of worsening left flank pain; she noticed malodorous urine 2–3 weeks prior and experienced left flank pain for several days before presentation. There was no personal or family history of underlying disease, but she was being treated for frequent by a local physician. The patient was admitted to the intensive care unit where she developed a fever (37.6°C) and tachycardia (heart rate 112 beats/min), with a blood pressure of 81/42 mmHg and respiratory rate of 20 breaths per min. A complete blood count revealed leukocytosis (1.57 × 10^9^ cells/L), thrombocytopenia (73 × 10^9^ cells/L), and anemia (hemoglobin 7.3 g/dL). Levels of C-reactive protein and procalcitonin were elevated (4.79 mg/dL and 294.64 ng/mL, respectively), and a prolonged prothrombin time was observed, with an internal normalized ratio of 2.28. Plasma D-dimer levels were elevated (286.4 μg/mL).

**Figure 1:**
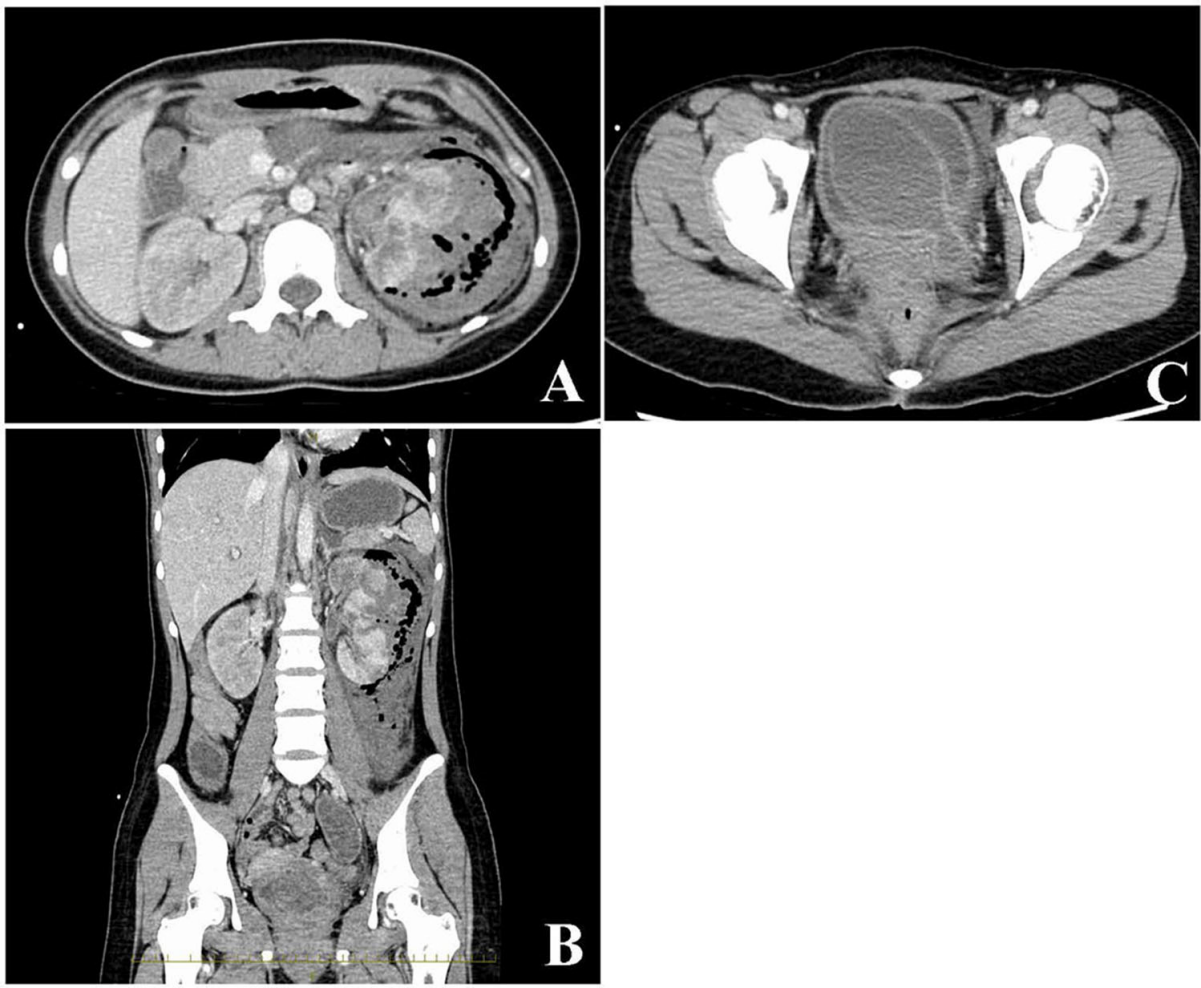
Radiological findings from an abdominal contrasted computed tomography scan. The damaged left kidney exhibits a poorly enhanced area and fluid and gas accumulation in the left retroperitoneal cavity **(A,B)**. A giant ureterocele evident in the bladder **(C)**.

Acute kidney injury (serum creatinine, 2.06 mg/dL) was detected. Serum lactate level (4.8 mmol/L) was high. The sequential organ failure assessment (SOFA) scores and quick SOFA scores were 6 and 2, respectively. The Japan Coma Scale score was 1, and Glasgow Coma Scale score was 14 (eye opening, 4; verbal response, 4; motor response, 6). Contrast-enhanced computed tomography revealed destructive left renal parenchyma replacement with a poor-contrasted area (Figure 1A) and fluid and gas accumulation from the perirenal region to the retroperitoneal cavity (Figure 1B). A giant ureterocele was observed in the left side of the bladder (Figure 1C). The patient was diagnosed with left emphysematous pyelonephritis with a congenital giant ureterocele.

The patient required noradrenalin administration (0.2 μg/kg/min). Her blood pressure remained normal, and surgical treatment, including transurethral ureterocele fenestration followed by urgent nephrectomy, was performed. Pus-containing urine spilled when the ureterocele was opened during fenestration, and a malodor was detected during transperitoneal open left nephrectomy. The excised outer portion of the left kidney had been dissolved by the infection and replaced with blood clots and necrotic tissue. The surgical specimen (weight, 354 g) consisted of the entire left kidney and ureter which was dissected near the bladder. The operative durations for the transurethral ureterocele fenestration and open left nephrectomy were 50 and 228 min, respectively. The estimated intraoperative blood loss was 1,424 mL, and no intraoperative complications occurred. Histopathological examination revealed neutrophils and bacteria in the perirenal fat, renal cortex, and medulla. Four bacterial species were detected in the urine culture, and three bacterial species were found in the blood culture before the administration of antibiotics. Bacteria detected in the urine sample were *A. schaalii, Actinomyces turicensis, Prevotella bergensis*, and *Prevotella disiens*.

**Figure 2:**
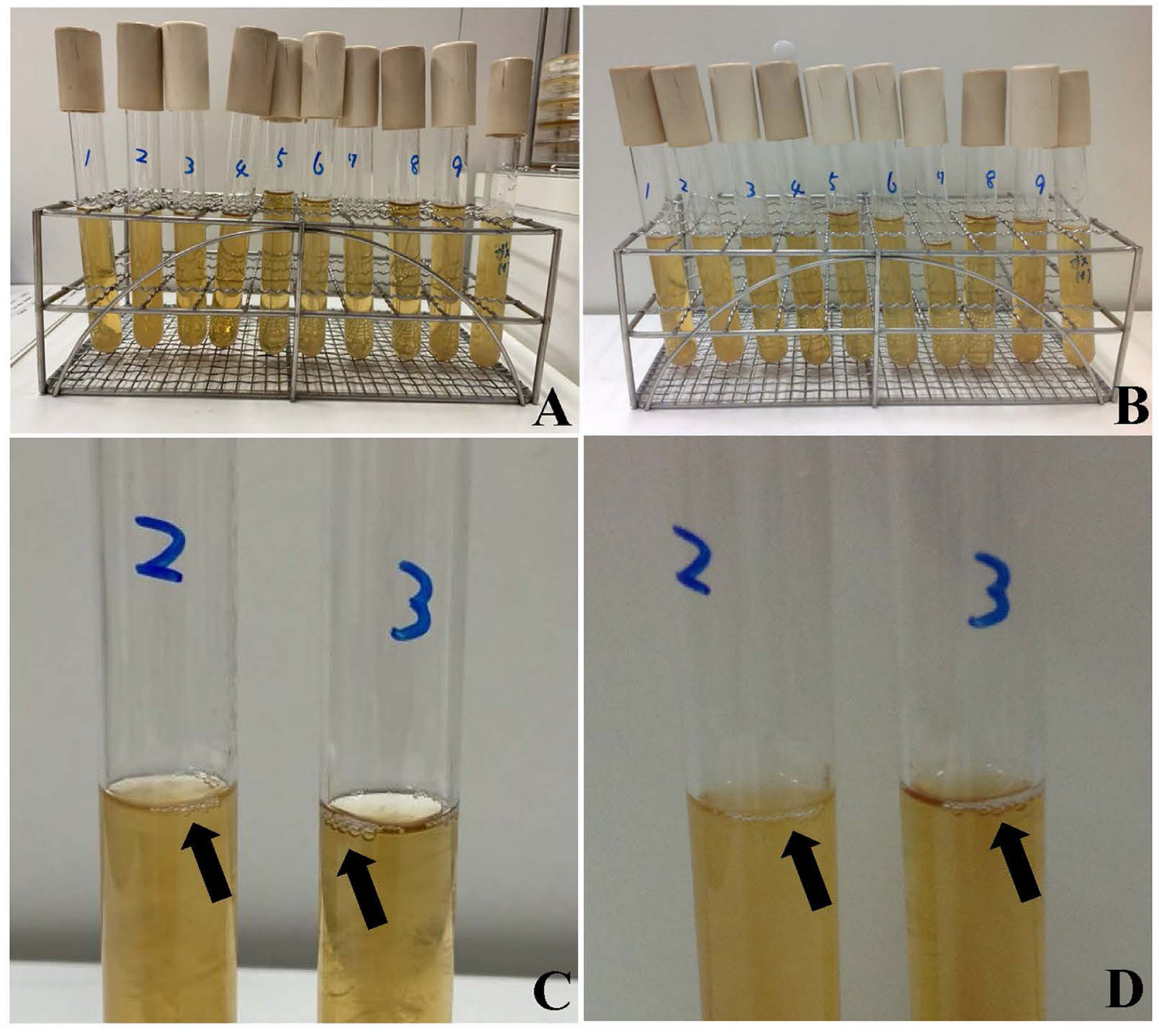
Bacteria detected in urine and blood samples were cultured in a pre-reduced anaerobically sterilized medium. Bacteria detected after 24 h **(A)** and after 48 h **(B)**. *Clostridium perfringens* was cultured for comparison of gas production. *Actinotignum schaalii* and *Peptoniphilus asaccharolyticus* produced gas in the KM media for 24 h **(C)**, 48 h **(D)**. Bacterial cultures from left to right are *Actinotignum schaalii* (1), *Actinotignum schaalii* (2), *Peptoniphilus asaccharolyticus* (3), *Actinomyces odontolyticus* (4), *Prevotella bergensis* (5), *Actinotignum schaalii* (6), *Actinomyces turicensis* (7), *Prevotella disiens* (8), *Actinomyces odontolyticus* (9), and *Clostridium perfringens*. Cultures 1–4 and 9 are from blood samples, with 1 and 2, and 4 and 9, cultured from independent blood samples. Cultures 5–8 are from urine samples.

Matrix-assisted laser desorption ionization time-of-flight mass spectrometry (MALDI-TOF MS) identified *A. schaalii, P. asaccharolyticus*, and *A. odontolyticus* in the blood culture as the inflammation-causing bacteria. All bacteria detected in urine and blood samples, along with *Clostridium perfringens*, which was used as the control for detecting gas production, were cultured using a pre-reduced anaerobically sterilized medium (Kyokuto Pharmaceutical Industrial), and the medium was observed after 24 and 48 h (Figures 2A,B). *A. schaalii* and *P. asaccharolyticus* were identified as the gas-producing bacteria in the blood culture (Figures 2C,D). Meropenem (1 g/body every 8 h) was initiated on admission and switched to ampicillin-sulbactam (4.5 g/body every 8 h) on postoperative day 4. The total course of antibiotic administration was 14 days. She had experienced wound pain, which decreased in intensity after ~1 week and her general condition improved. She was discharged on day 17 of hospitalization. The patient had no recurrent urinary tract infections after discharge. No postoperative complications were noted 6 months after hospital discharge.

## Discussion

EPN is a relatively rare but rapidly progressive urinary tract infection characterized by gas accumulation in the renal parenchyma, collecting ducts, and perinephric tissue (1, 2). In particular, EPN is extremely rare in children (12). To the best of our knowledge, this is the first reported case of severe EPN with a ureterocele in a pediatric patient with *A. schaalii, P. asaccharolyticus*, and *A. odontolyticus* as the causative bacteria.

The first case of EPN in adults was reported in 1898 (13), and the first case of EPN in a pediatric patient was reported in 1985 (6). Several key factors related to the development of EPN have been identified, including high renal glucose levels, urinary tract obstruction, decreased immunity, and presence of gas-producing bacteria (4, 14). Approximately 90% of patients who develop EPN (especially female patients) have diabetes mellitus (2). Conversely, none of the reported cases of EPN in pediatric patients have indicated diabetes mellitus as a comorbidity (Table 1). Obstructive uropathy, neurogenic bladder, kidney stones, and impaired host immunity may be risk factors for pediatric EPN (12).

**Table 1:** Review of the literature regarding emphysematous pyelonephritis in the pediatric age group.

	**Sex**	**Age**	**Diabetes**	**Risk factors**	**Pathogen isolated**	**Treatment**	**Outcome**
Pode et al. (6)	F	10 years	No	Neurogenic bladder	*P. mirabilis, E. coli*	Antibiotics+PNS	Alive
Fernandes et al. (7)	M	6 years	No	Ureteropelvic junction obstruction	*Not done*	Not done	Alive
Al-Makadma et al. (8)	M	12 months	No	Neurogenic bladder	*E. coli*	Antibiotics	Alive
Siddique and Seikaly (9)	F	3 months	No	Obstruction due to ectopic right ureter	*Enterobacter cloaca*	Antibiotics	Alive
Ambaram et al. (11)	M	9 months	No	Not done	*E. coli*	PNS	Dead
Ambaram et al. (11)	F	34 months	No	Acquired immunodeficiency	*E. coli*	Antibiotics + PNS + LN	Alive
Gross and Ford (10)	F	4 years	No	Renal stone	*E. coli*	Antibiotics	Alive
Girgenti et al. (12)	M	23 months	No	Nephrourological congenital malformation surgery	*E. coli*	Antibiotics	Alive

*PNS, Percutaneous drainage; LN, Laparoscopic nephrectomy*.

The most common EPN-causing pathogen is *E. coli*, accounting for ~70% of cases; other pathogens include *Proteus mirabilis, Klebsiella pneumoniae*, group D *Streptococcus*, coagulase-negative *Staphylococcus*, and *Enterobacteriaceae* (2, 15). The causative pathogens of pediatric EPN are largely unknown; previous reports have indicated that *Enterobacteriaceae* and *E. coli* are common (6–12). In our case, several causative pathogens were identified. It is rare for *A. schaalii* (formerly *Actinobaculum schaalii*) to cause urinary tract infections. *A. schaalii* is a facultative, anaerobic, gram-positive, rod-shaped bacterium. The requirement of blood agar media incubated for 48 h under 5% CO_2_, or in anaerobiosis, to detect *A. schaalii* in urine or blood samples may cause it to be under-detected because most clinical microbiological laboratories do not routinely culture urine samples under anaerobic conditions (16, 17).

*A. schaalii* has been reported in urine samples of children aged < 4 years and in older individuals aged > 60 years (18, 19) and is considered a part of the normal urogenital flora (18–20). We used MALDI-TOF MS analysis and performed 16S rRNA gene sequencing, which are reportedly more accurate than polymerase chain reaction in identifying *A. schaalii* (17). To the best of our knowledge, the ability of *A. schaalii* to produce gas has not been reported. We observed production of small quantities of gas by *A. schaalii*. In the patient in the present report, there was no history of diabetes mellitus, which supports the conclusion that the bacteria detected in the urine and blood samples produced the gas in the kidney.

In this case, *A. schaalii* was resistant to antibiotic therapy. *A. schaalii* is frequently resistant to trimethoprim–sulfamethoxazole and second-generation quinolones (norfloxacin and ciprofloxacin) (17), so careful selection of antibiotics is needed in urinary tract infections caused by gram-positive, rod-shaped bacteria including *A. schaalii*, with β-lactams as the first-line treatment.

Although EPN is a life-threatening illness with a mortality rate of 12.5–50% (3, 12), no guidelines have been established for the optimal management of patients with EPN. The main treatment options are percutaneous drainage (PCD) and medical management with or without stenting of the urinary tract (12). Medical management of EPN requires fluid and electrolyte replacement, correction of acid-base imbalances, and antibiotic therapy. Inotropes are also required in some patients (12). Urgent nephrectomy is the standard surgical treatment, though recent surgical strategies suggest that an initial nephron-sparing approach with PCD followed by elective nephrectomy at a later time is effective (4, 12). Nephrectomy has been described in only one pediatric patient (6–12). In this patient, urgent nephrectomy was recommended because of the poor general condition and lack of response to conservative treatment, and good results were obtained. Most cases of EPN in children are treated conservatively; however, given the young age of our patient, PCD may have been preferable to preserve renal function. However, the indications for nephrectomy for pediatric EPN require further study and accumulation of more cases.

## Data Availability Statement

The raw data supporting the conclusions of this article will be made available by the authors, without undue reservation.

## Ethics Statement

Written informed consent was obtained from the minor(s)' legal guardian/next of kin for the publication of any potentially identifiable images or data included in this article.

## Author Contributions

All authors listed have made a substantial, direct, and intellectual contribution to the work and approved it for publication.

## Conflict of Interest

The authors declare that the research was conducted in the absence of any commercial or financial relationships that could be construed as a potential conflict of interest.

## Publisher's Note

All claims expressed in this article are solely those of the authors and do not necessarily represent those of their affiliated organizations, or those of the publisher, the editors and the reviewers. Any product that may be evaluated in this article, or claim that may be made by its manufacturer, is not guaranteed or endorsed by the publisher.
